# Sendai F/HN pseudotyped lentiviral vector transduces human ciliated and non-ciliated airway cells using α 2,3 sialylated receptors

**DOI:** 10.1016/j.omtm.2022.07.002

**Published:** 2022-07-06

**Authors:** Rosie J. Munday, Tiziana Coradin, Rachael Nimmo, Yatish Lad, Stephen C. Hyde, Kyriacos Mitrophanos, Deborah R. Gill

**Affiliations:** 1 Gene Medicine Research Group, Nuffield Division of Clinical Laboratory Sciences, Radcliffe Department of Medicine, John Radcliffe Hospital (Level 4), University of Oxford, Oxford OX3 9DU, UK; 2 Oxford Biomedica (UK) Ltd., Oxford OX4 6LT, UK

**Keywords:** lentiviral vector, pseudotype, human airway, Sendai virus, avian influenza virus, sialic acid, lectin, hemagglutinin, air-liquid interface, sialidase

## Abstract

A lentiviral vector (LV) pseudotype derived from the fusion (F) and hemagglutinin-neuraminidase (HN) glycoproteins of a murine respirovirus (Sendai virus) facilitates efficient targeting of murine lung *in vivo*. Since targeting of the human lung will depend upon the availability and distribution of receptors used by F/HN, we investigated transduction of primary human airway cells differentiated at the air-liquid interface (ALI). We observed targeting of human basal, ciliated, goblet, and club cells, and using a combination of sialidase enzymes and lectins, we showed that transduction is dependent on the availability of sialylated glycans, including α2,3 sialylated N-acetyllactosamine (LacNAc). Transduction via F/HN was 300-fold more efficient than another hemagglutinin-based LV pseudotype derived from influenza fowl plague virus (HA Rostock), despite similar efficiency reported in murine airways *in vivo*. Using specific glycans to inhibit hemagglutination, we showed this could be due to a greater affinity of F/HN for α2,3 sialylated LacNAc. Overall, these results highlight the importance of identifying the receptors used in animal and cell-culture models to predict performance in the human airways. Given the reported prevalence of α2,3 sialylated LacNAc on human pulmonary cells, these results support the suitability of the F/HN pseudotype for human lung gene therapy applications.

## Graphical abstract

**Figure:**
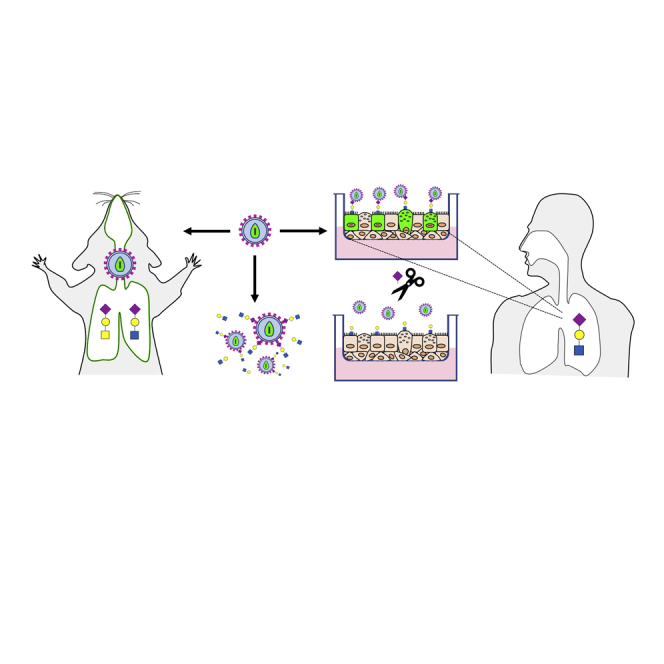

## Teaser

Munday et al. investigate the potential for human airway cell targeting by lentiviral vectors pseudotyped with Sendai F/HN glycoproteins. Transduction of ciliated and non-ciliated airway cells was dependent on the availability of sialylated glycans and efficient use of α2,3 sialylated N-acetyllactosamine.

## Introduction

Recombinant lentiviral vectors (LVs) have been extensively developed for clinical gene therapy and offer multiple advantages, including a relatively large transgene packaging capacity, the ability to transduce both dividing and non-dividing cells, and genome integration leading to long-term transgene expression. In addition, LVs can be pseudotyped to increase efficiency for targeting of specific cells.

Optimized over 2 decades, LVs are now widely accepted as successful vehicles for gene introduction to cells *ex vivo*, treating both genetic and acquired diseases, such as X-linked adrenoleukodystrophy,^1^ and cancer, such as acute lymphoblastic leukemia (ALL), by engineering a patient’s own T cells to turn them into chimeric antigen receptor (CAR)-T cells, which are able to recognize and kill cancer cells.^2^ These applications use the glycoprotein from vesicular stomatitis virus (VSV-G) as a pseudotype to facilitate transduction of a broad range of mammalian cell types; however, for efficient targeting of some tissues and cell types, more specific pseudotypes are required. For example, polarized lung epithelial cells are refractory to LV pseudotyped with VSV-G,^3^ likely due to the basolateral distribution of VSV-G receptors on polarized epithelia, and so alternative apical lung-targeting pseudotypes are being investigated.

Recombinant simian immunodeficiency virus (rSIV) pseudotyped with the murine Sendai virus fusion (F) and hemagglutinin-neuraminidase (HN) glycoproteins has been developed (rSIV.F/HN), transducing murine airways and lung *in vivo* without the need for adjuncts.^4^ This property of rSIV.F/HN has been shown to facilitate murine lung expression of alpha-1 anti-trypsin (AAT) for treatment of AAT deficiency^5^ and for secretion of antibodies against infection with influenza,^6^ respiratory syncytial virus,^7^ and severe acute respiratory syndrome coronavirus 2 (SARS-CoV-2).^8^ Following the success of the rSIV.F/HN platform in mouse models and the ability to transduce multiple murine lung cell types, this LV is being developed for treatment of respiratory diseases, such as cystic fibrosis (CF).^4^ However, poor understanding of the specific receptors used by the F/HN pseudotype leads to uncertainty about the performance of this pseudotype in the human lung as well as the relevance of different animal models in predicting this.

The HN glycoprotein from Sendai virus recognizes and binds sialylated glycans on the cell surface, acting as the fundamental first step in virus entry. This binding is followed by fusion of the virus envelope and host cell membrane mediated by the F glycoprotein.^9^ Glycans are sialylated with a glycosidic link between the second carbon atom of sialic acid and (usually) the third (α2,3) or sixth (α2,6) carbon of a galactose ring. The dependence of Sendai virus on the availability of α2,3 sialylation to mediate infection^10^ is also thought to underpin the infection of ciliated cells in murine tracheal epithelial cells in culture.^11^ These results, however, are in contrast with rSIV.F/HN-mediated gene delivery to both ciliated and non-ciliated murine airway cells *in vivo*; this raises questions over the ability of cultured cells to model receptor availability *in vivo* and also how well native Sendai virus infectivity might predict the performance of the F/HN pseudotype. Interestingly, another LV pseudotype (HA Rostock), which incorporates influenza hemagglutinin (HA) from fowl plague virus H7N1 (A/FPV/Rostock/8/1934), shows similarly efficient transduction of murine airway *in vivo*,^12^ as well as a preference for targeting ciliated cells in murine tracheal cultures;^13^ it is also predicted to use α2,3 sialylated receptors.^14^

**Figure 1:**
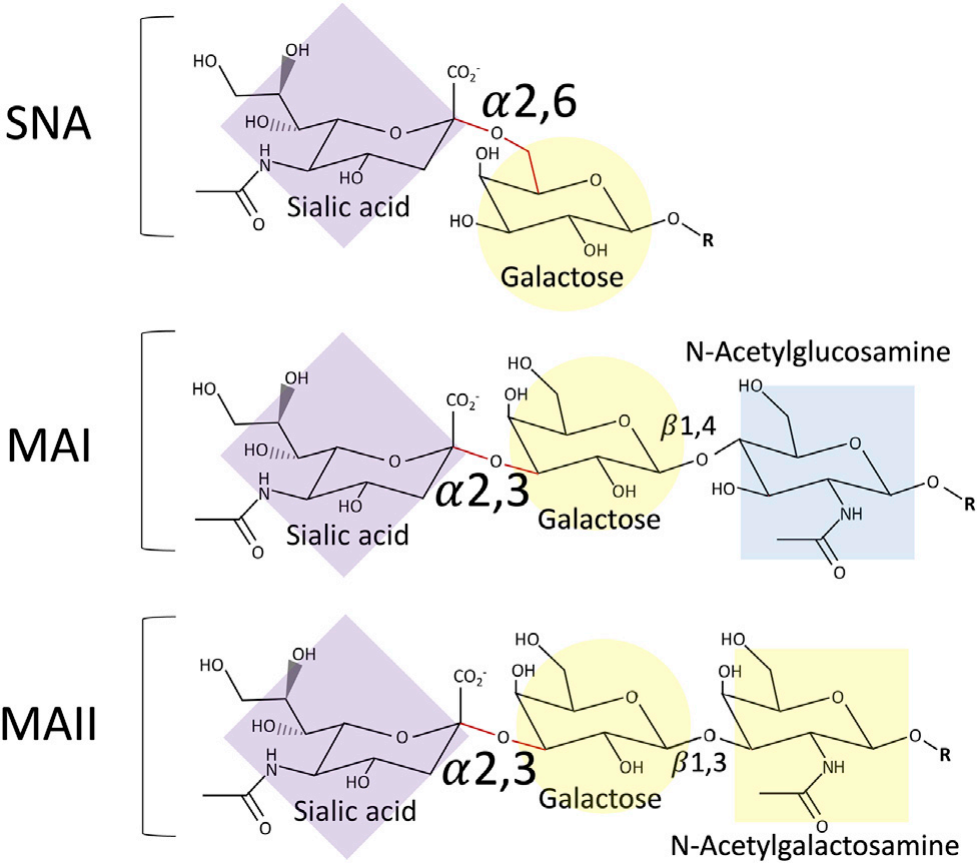
Sialylated glycan subtypes detected by specific lectin isolates Sialic acid (N-acetylneuraminic acid) is connected via α2,6 or α2,3 glycosidic linkage to the galactose of a glycan. Sialylated glycans with α2,6 glycosidic linkages are recognized by lectins isolated from *Sambucus nigra* agglutinin (SNA), whereas α2,3 linkages are recognized by *Maackia amurensis* (MAI or MAII) lectins. The consensus saccharide sequence bound by each lectin (reviewed by Geisler and Jarvis^15^) and its structure is shown. Each monosaccharide is shaded following the symbol nomenclature for glycans (SNFG) system;^16^ sialic acid (purple diamond); galactose (yellow circle); N-acetylglucosamine (blue square), and N-acetylgalactosamine (yellow square).

Lectins that bind specific sialylated glycans are commonly used to stain and distinguish between the subtypes available. Although a lectin isolated from *Sambucus nigra* agglutinin (SNA) will detect any α2,6 linkage, the lectins isolated from *Maackia amurensis*, MAI or MAII, are more specific, detecting α2,3 sialylation of galactose linked to N-acetylglucosamine or N-acetylgalactosamine, respectively (Figure 1).^15^ While using a mixture of MAI and MAII lectins enables detection of both α2,3 sialylation subtypes, the use of each isolate individually can reveal differences in the availability of these subtypes, such as in human airway and lung.^17^ This is important when characterizing receptor availability, given the differences between viruses, such as murine Sendai, in ability to bind these subtypes.^18^ In the murine lung, although α2,3 is generally more available than α2,6,^11^^,^^19^ the availability of the specific α2,3 subtype bound by MAI, which is also the only α2,3 subtype identified in human airway (herein referred to as human α2,3), is unknown.

Cultures of human bronchial epithelial cells (HBECs) differentiated at an air-liquid interface (ALI) are a well-established model of polarized, pseudostratified mucociliary epithelium,^20^ which have been used to investigate receptors for transduction on the apical and basolateral surfaces.^21^ In contrast to murine models, human ALI cultures express α2,6 residues in common with human bronchi^11^ and thus are potentially a more useful model of receptor availability in the human airways. However, the ability of ALI cultures to model the specific α2,3 subtypes found in human airway is unknown. Furthermore, there are conflicting reports of co-localization of lectin staining and immunohistochemistry (IHC) for specific lung cell types,^11^^,^^22^ which highlight the need for further investigation. These experimental discrepancies may be due to differences in protocols for lectin staining and ALI culture maintenance. For instance, the culture of epithelial cells sourced from smaller (bronchiolar) versus larger (bronchial) airways can result in a different cell-type availability,^23^ which could readily affect the sialylated glycans expressed.

Apical delivery of rSIV.F/HN to human bronchial ALI cultures has been shown to result in abundant (luciferase) transgene expression,^24^^,^^25^ although neither the receptors used for transduction nor the cell types transduced were identified. Here, we confirm that, in contrast to murine airway, human ALI cultures can model the α2,3 subtype availability found in human airways. We show that F/HN mediates transduction of all major human airway epithelial cell types. By using pre-treatment of cultures with sialidase to cleave sialylated linkages or incubation with lectins to block receptor binding, we confirm that F/HN transduction requires sialylated glycans, with the human α2,3 subtype as the primary receptor used. Further, through inhibition of hemagglutination, we identified that F/HN has a greater affinity to bind the human α2,3 subtype of sialylated glycans, compared with the HA Rostock LV pseudotype. Based on these findings, we predict that the F/HN pseudotype will facilitate efficient targeting of LVs for a range of human lung gene therapy applications.

## Results

### Modeling the human airway epithelium using ALI cultures of human airway epithelial cells

**Figure 2:**
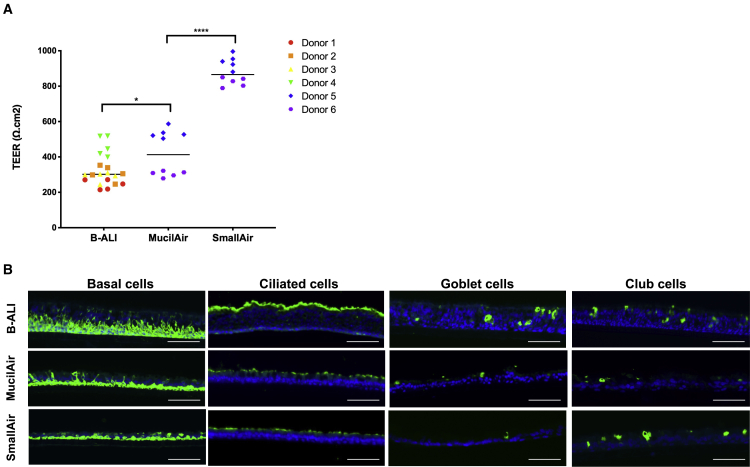
Characterization of three types of primary human ALI culture Human air-liquid interface (ALI) cultures generated from human bronchial epithelial cells (HBECs) in house (B-ALI) or obtained fully differentiated (MucilAir or SmallAir) were characterized. (A) The trans-epithelial electrical resistance (TEER) was measured for each ALI culture type across multiple founding cell donors. Each data point represents a biological replicate; the bar represents the median. ∗p = 0.0244 (B-ALI versus MucilAir) and ∗∗∗∗p < 0.0001 (MucilAir versus SmallAir) as determined using Mann-Whitney U test. (B) Immunohistochemistry of ALI culture cryosections using cell-type-specific antibodies to detect basal (cytokeratin 5), ciliated (β-tubulin), goblet (mucin 5AC), and club cells (CC10) shown in green. Images are representative; nuclei are counter-stained blue (DAPI); scale bars represent 100 μm.

Airway ALI cultures contain at least three of the major cell types found in proximal human airways, including basal, goblet, and ciliated cells, the abundance of which can be influenced by the culture materials and methods. We therefore assessed how three different human ALI cultures mimicked the human airway epithelium. Human bronchial epithelial cells (HBECs) (n = 4 donors) obtained from a commercial source (Lonza, Basel, Switzerland) were expanded and differentiated at an ALI using an established protocol (STEMCELL Technologies) to generate bronchial ALI (B-ALI) cultures. We also assessed fully differentiated ALI cultures, derived from either bronchial (MucilAir) or bronchiolar (SmallAir) epithelial cells, commercially supplied and maintained using proprietary media as directed (Epithelix Sarl, Geneva, Switzerland). For each culture type, the trans-epithelial electrical resistance (TEER) of the polarized epithelium was measured (Figure 2A). An ∼1.3-fold (significant) difference in TEER (p *=* 0.0244) was detected between the B-ALI and MucilAir cultures, despite derivation of both from bronchial epithelial cells. Interestingly, we observed an even greater (∼2-fold) difference (p *<* 0.0001) in TEER between the commercially supplied MucilAir and SmallAir cultures.

To characterize the cellular composition of the three types of human ALI culture, cryosections were prepared and IHC performed using antibodies against cell-specific markers of basal (cytokeratin 5), ciliated (β-tubulin), goblet (Mucin 5AC), and club (CC10) cells. Figure 2B shows representative images of IHC staining (green), showing that basal and ciliated cells were present in all three ALI culture types, with greatest abundance in B-ALI cultures. Smaller numbers of goblet and club cells were also detected in all three culture types, with goblet cells observed only rarely in SmallAir cultures, in agreement with published reports from others.^23^ There were no obvious differences in the abundance of club cells between any of the cultures, despite a reported absence of club cells from MucilAir cultures previously.^23^

### F/HN pseudotyped LV transduces all ALI culture types but with different efficiencies

**Figure 3:**
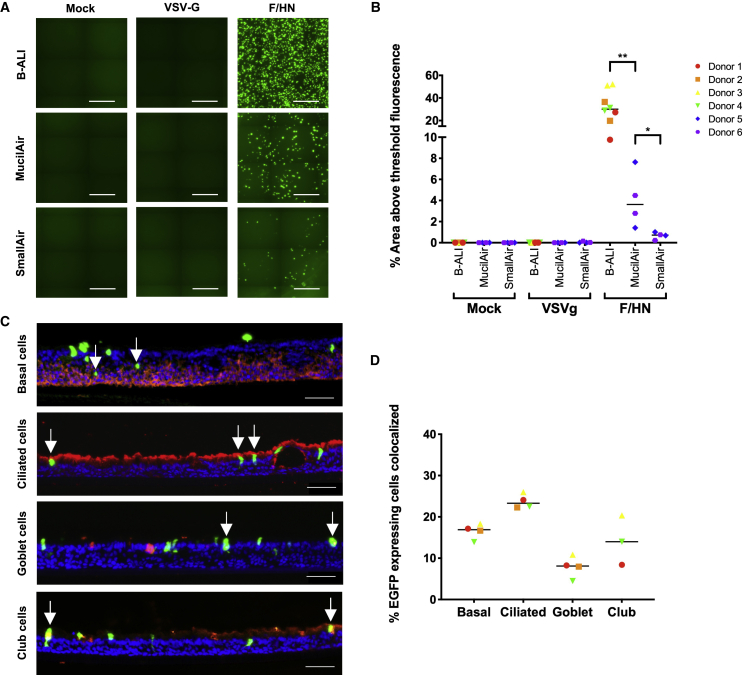
Transduction of different ALI culture types with F/HN pseudotyped LV (A) Native EGFP detection from ALI cultures 14 days after apical delivery of 7.5 × 10^7^ transducing units (TUs) of LV.F/HN or LV.VSV-G expressing EGFP or mock-treated (buffer only). Representative stitched center-of-transwell images are shown (B-ALI, n = 4 donors; MucilAir and SmallAir, n = 2 donors; n = 2 biological replicates for each); scale bars represent 500 μm. (B) Quantification of EGFP (as percentage area above threshold fluorescence) from ALI cultures transduced or mock treated (buffer only) is shown. Each symbol represents a biological replicate, each shape represents a single donor, and the bar represents the median. ∗∗p = 0.004 (LV.F/HN-treated B-ALI versus MucilAir) and ∗p = 0.0286 (MucilAir vs SmallAir), as determined using Mann-Whitney U test. (C) Representative images of transwell cryosections show cell types transduced by LV.F/HN in B-ALI cultures, identified by co-localization (white arrows) of EGFP (green) with cell-type-specific antibodies (shown in red) to detect: basal (cytokeratin 5), ciliated (β-tubulin), goblet (mucin 5AC), and club (CC10) cells. Donor 1 is shown; nuclei are stained blue (DAPI); scale bars represent 50 μm. (D) Quantification of cell types expressing EGFP following transduction of B-ALI cultures with LV.F/HN is shown. Co-localization of EGFP and cell-specific markers was calculated from a minimum count of 100 EGFP-positive cells per B-ALI culture donor (n = 4) for each cell type. Each shape represents the cell donor; the bar represents the median. No club cells were identified in donor 2.

We wished to compare transduction efficiency in the three ALI culture types. Recombinant HIV vectors pseudotyped with either F/HN (LV.F/HN) or VSV-G (LV.VSV-G) each expressing EGFP were produced. Approximately 7.5 × 10^7^ transducing units (TUs) of LV.F/HN or LV.VSV-G were administered to the apical surface of the ALI cultures and compared with mock-treated (buffer only) control cultures. Native EGFP was measured 14 days after delivery. Representative images (Figure 3A) show that no EGFP fluorescence was detected for cultures treated with LV.VSV-G or mock treated with buffer only (control). This lack of transduction is consistent with published studies showing that human ALI cultures are resistant to transduction from the apical surface by VSV-G pseudotyped LV.^26^ Low-level transduction of cells at the edge of the ALI cultures was observed, confirming that the LV.VSV-G preparation was functional and able to transduce a small number of cells with reduced epithelial cell contact at the edge of the transwell (Figure S1).

LV pseudotyped with F/HN transduced all three ALI culture types but transduced B-ALI cultures most efficiently (∼8-fold higher; p = 0.004 compared with MucilAir) (Figure 3B). Measurement of the % area of EGFP fluorescence showed LV.F/HN transduction of up to 52% in individual B-ALI cultures derived from bronchial cells, with a maximum of only 8% in MucilAir cultures, also derived from bronchial cells (p = 0.004). In SmallAir cultures, <1% of the area was positive, significantly lower than MucilAir cultures despite being derived from the same donors (p = 0.0286). To identify the cell types transduced by F/HN, cryosections were stained (red) with cell-type-specific antibodies (Figure 3C). Co-localization of EGFP and antibody staining (white arrows) revealed that LV.F/HN could transduce all four cell types identified in the ALI cultures. Quantification of the % of EGFP-positive cells co-localizing with each cell-type-specific antibody was challenging, due to the small numbers detected in cryosections, but there was a trend for more LV.F/HN-transduced cells to be identified as ciliated cells (Figure 3D). Interestingly, the ciliated cell type is more abundant in B-ALI cultures compared with MucilAir and SmallAir cultures (Figure 2B) and could easily explain the more efficient LV.F/HN transduction of B-ALI cultures compared with other ALI culture types.

### Human ALI cultures model the availability of sialylated glycans in human airway

**Figure 4:**
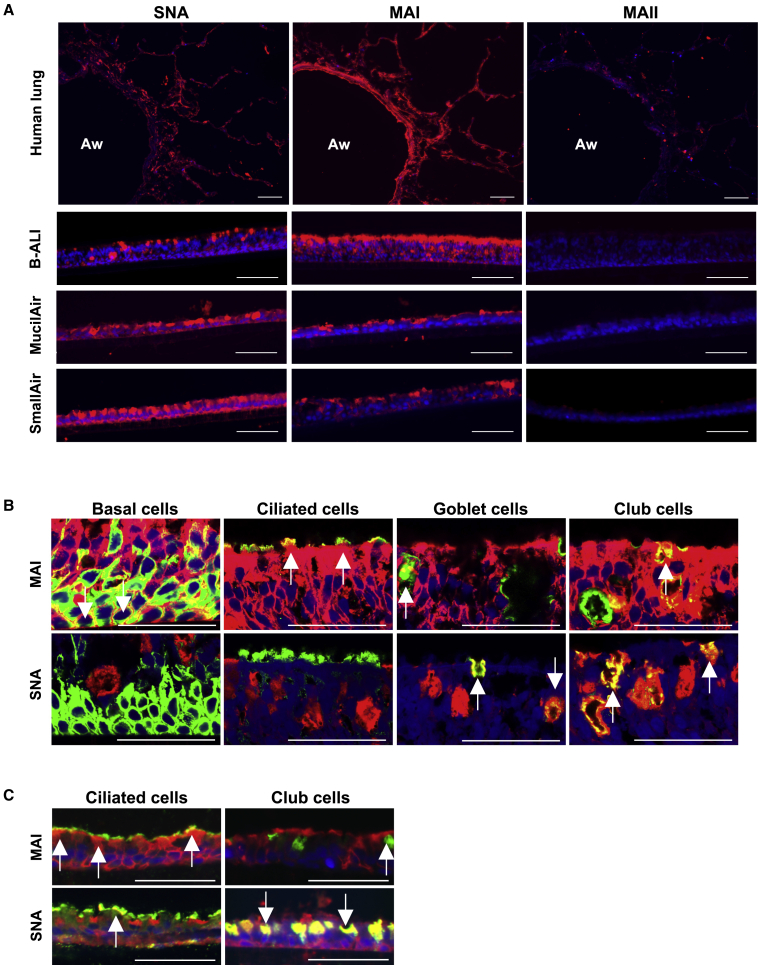
Characterization of the availability of sialylated glycans and their distribution between cell types (A) Lectin staining (red) of human lung and ALI culture cryosections performed in parallel. Representative images are shown; nuclei are stained blue (DAPI); Aw, airway; scale bars represent 100 μm. Cryosections pre-treated using Sialidase from *Arthrobacter ureafaciens* (Sialidase A) to cleave sialic acid were also stained in parallel to confirm staining is specific to sialylated glycans (see Figure S2 for representative images, together with staining of murine lung cryosections). (B and C) Dual lectin staining (red) and immunohistochemistry (green) of cryosections of B-ALI (B) and MucilAir (C) cultures using cell-type-specific antibodies for basal (cytokeratin 5), ciliated (β-tubulin), goblet (mucin 5AC), and club cells (CC10). Cells labeled by immunohistochemistry that co-localize with lectin staining appear yellow (white arrows); nuclei are stained blue (DAPI); scale bars represent 50 μm. To confirm absence of SNA-stained basal and ciliated cells in B-ALI cultures, images were captured using a Zeiss LSM 780 inverted confocal microscope (63× oil immersion) to achieve maximum resolution (B). Stained MucilAir culture cryosections were imaged using a widefield fluorescence microscope (EVOS FL Auto 2) (C).

To investigate the ability of these ALI cultures to model the availability of sialylated glycans in human airway, lectin staining was performed on cryosections of the human ALI cultures, together with human-lung-tissue-containing airways (Figure 4A). In human lung, widespread MAI and SNA lectin staining confirmed an abundance of human α2,3 subtype and α2,6 sialylated glycans, respectively, throughout the airway and parenchyma. This was in contrast with the α2,3 subtype bound by MAII lectin, which showed only minimal staining. This overall pattern of lectin staining (red; MAI > SNA > MAII) in human lung agrees with published results using these lectins^17^^,^^27^ and with the staining predicted from mass spectrophotometry of isolated glycans.^27^^,^^28^ Figure 4A also reveals that all three human ALI culture types model the α2,6 and α2,3 subtype-specific availability (strong SNA and MAI staining but no MAII staining). Interestingly, B-ALI cultures most closely reflected the relative abundance of human α2,3 subtype and α2,6 glycans (MAI > SNA) observed in the human airway. These results indicate that all ALI culture types are a suitable model of the human airway in which to assess transduction mediated by sialylated glycan receptors. Importantly, this finding is in contrast with the murine lung model, where the absence of SNA staining in murine airway confirms a lack of α2,6 residues (Figure S2).^11^ Furthermore, in addition to diffuse MAI staining of the human α2,3 subtype in murine lung, a similar staining pattern was also observed using MAII (Figure S2), confirming an abundance of the α2,3 sialylated glycan subtype that is *not* available in the human airway.^17^

### Sialylated glycan subtypes are enriched on specific cell types

To investigate whether certain sialylated glycan subtypes are specific to certain cell types, dual staining with lectins (red) and cell-type-specific antibodies (green) was performed to determine co-localization (yellow; white arrows; Figures 4B and 4C). Confocal images of B-ALI cultures showed that MAI staining (for the human α2,3 subtype) could be detected on all cell types, with greatest abundance on basal and ciliated cells; however, not every goblet and club cell co-localized with MAI lectin (Figure 4B, representative images). Conversely, α2,6 staining with SNA was observed on all goblet and club cells (Figure 4B), with minimal detection on ciliated or basal cells in B-ALI cultures. Results with MucilAir cultures (Figure 4C) were similar, showing enrichment of MAI staining on ciliated cells, and SNA staining concentrated on non-ciliated (club) cells. However, the abundance and cell type specificity of the subtypes varied between ALI culture types (e.g., SNA staining of ciliated and [presumed] basal cells was found only in MucilAir cultures); this suggests that the ALI culture protocol may also significantly influence staining patterns.

### F/HN preferentially uses human α2,3 sialylated glycans to transduce ALI cultures

**Figure 5:**
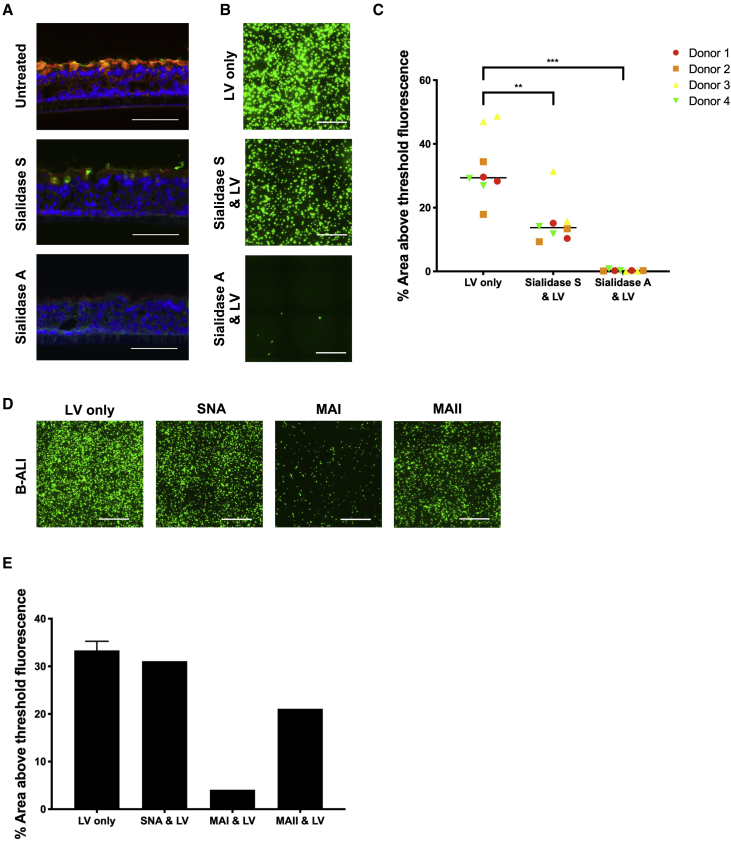
The effect of pre-treatment of B-ALI cultures with Sialidase or lectin prior to transduction with F/HN pseudotyped LV (A) B-ALI cultures were pre-treated with Sialidase A or Sialidase S for 1 h or left untreated as controls and fixed for staining of cryosections using lectins MAI (red) and SNA (green). Representative images are shown (donor 1); nuclei are stained blue; scale bars represent 100 μm. (B) B-ALI cultures pre-treated with Sialidase A or Sialidase S were transduced with LV.F/HN expressing EGFP (7.5 × 10^7^ TUs) or left untreated (no LV) and imaged 14 days later. Representative stitched center-of-transwell images (donor 3) are shown; scale bars represent 500 μm. (C) EGFP fluorescence from transduced B-ALI cultures was quantified. Each symbol represents a biological replicate; each symbol represents a single donor; bar represents the median. LV only versus Sialidase A and LV, ∗∗∗p = 0.0002 (F/HN); LV only versus Sialidase S and LV, ∗∗p = 0.003 (F/HN). Significance was determined using Mann-Whitney U test; ns, not significant. (D and E) B-ALI cultures derived from a single HBEC donor were pre-treated with lectins (100 μg/mL) for 2 h or left untreated as controls (LV only) and then transduced with LV.F/HN (7.5 × 10^7^ TUs) expressing EGFP and imaged 14 days later. (D) Representative stitched center-of-transwell images are shown; scale bars represent 500 μm. (E) Quantified EGFP fluorescence from transduced B-ALI cultures. Error bar represents standard deviation of n = 2 (LV only) control replicates; bar represents mean. Remaining bars represent a single transwell.

We investigated whether LV.F/HN uses the human α2,3 subtype of sialylated glycans, most abundant on B-ALI cultures, as receptors for transduction. We used Sialidase S (from *Streptococcus pneumoniae*), which cleaves α2,3 sialic acid linkages, and Sialidase A (from *Arthrobacter ureafaciens*), which cleaves both α2,3 and α2,6 linkages.^29^ B-ALI cultures were treated apically with Sialidase A or Sialidase S for 1 h (or left untreated as controls) and then fixed for staining with lectins MAI (red) and SNA (green). As expected, treatment with Sialidase S reduced MAI (red) staining and cultures treated with Sialidase A showed almost no staining with either lectin, compared with untreated cultures (Figure 5A). In parallel, B-ALI cultures treated with Sialidase enzymes were transduced with LV.F/HN expressing EGFP (7.5 × 10^7^ TUs) and imaged 14 days later. The number of transduced EGFP-positive cells was reduced after treatment with Sialidase S and abolished by Sialidase A (Figure 5B). EGFP fluorescence decreased from 33% to 15% (p = 0.003) after cleavage of α2,3; however, EGFP detection was essentially abolished (<1%; p = 0.0002) after treatment with Sialidase A (Figure 5C). This indicates that LV.F/HN uses α2,3 sialylated glycans to transduce B-ALI cultures, but sialylated glycans that remain following Sialidase S treatment (potentially α2,6) are also used. To investigate the relative use of α2,3 versus α2,6 glycans as receptors, we compared the ability of lectins to block transduction of B-ALI cultures. Lectins were applied to the apical surface and incubated at 37°C for 2 h to allow binding of sialylated glycan substrates prior to the addition of LV.F/HN (7.5 × 10^7^ TUs). Transduction was inhibited (∼8-fold) only by the addition of MAI (Figures 5D and 5E), indicating that LV.F/HN predominantly uses the human α2,3 subtype to transduce B-ALI cultures.

### The relative affinity of F/HN for human α2,3 and α2,6 sialylated glycans

**Figure 6:**
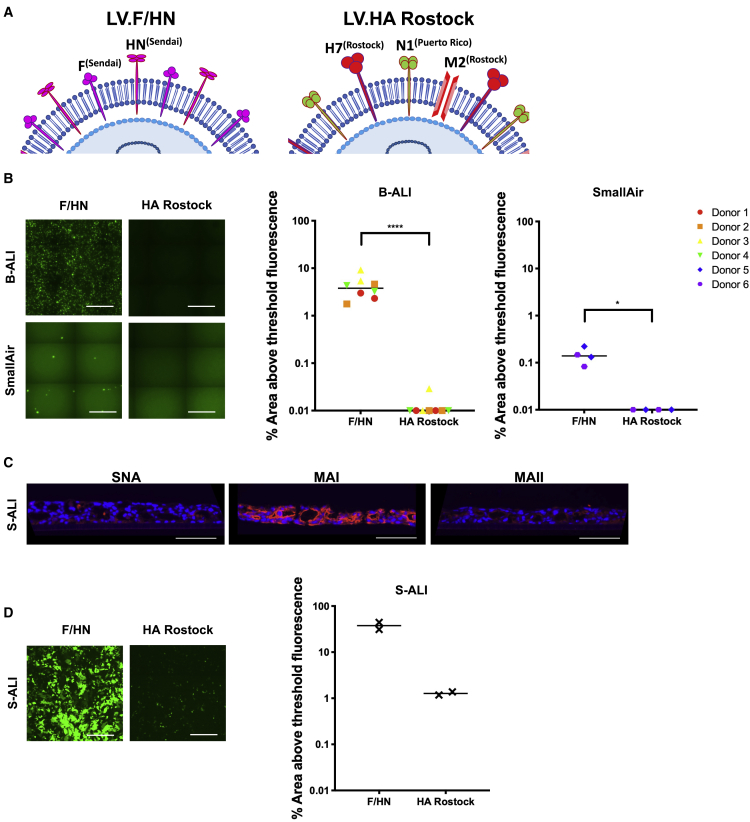
The relative efficiency of transduction of human ALI cultures with F/HN and HA Rostock pseudotyped LV (A) Diagrammatic representations of Lentiviral vector pseudotypes: LV.F/HN comprises fusion (F^(Sendai)^) and hemagglutinin-neuraminidase (HN^(Sendai)^) glycoproteins derived from Sendai virus; LV.HA Rostock comprises hemagglutinin glycoprotein from H7N1 A/FPV/Rostock/8/1934 (H7^(Rostock)^). The LV.HA Rostock influenza pseudotype is supplemented with expression of influenza M2 glycoprotein (proton channel) from the Rostock strain (M2^(Rostock)^) and an influenza neuraminidase glycoprotein from H1N1 A/Puerto Rico/8/1934 N1) (N1^(Puerto Rico)^). (B) LV.F/HN or LV.HA Rostock, each expressing EGFP, was administered to the apical surface of B-ALI (7.5 × 10^6^ TUs per transwell) and SmallAir cultures (4.7 × 10^6^ TUs per transwell) and examined 14 days later. Detection of native EGFP in representative, stitched center-of-transwell images is shown. Scale bars represent 500 μm. EGFP was also quantified from LV transduced cultures of B-ALI (∗∗∗∗p < 0.0001; LV.F/HN versus LV.HA Rostock) or SmallAir (∗p = 0.0245; LV.F/HN versus LV.HA Rostock). Each symbol represents a biological replicate; each shape represents a single donor; bar represents the median. Significance was determined using Kruskal-Wallis test followed by Dunn’s multiple comparison test. (C and D) Surfactant ALI (S-ALI) cultures were cryosectioned and stained with lectins to determine distribution of sialylated glycans (C) or transduced with LV.F/HN or LV.HA Rostock expressing EGFP (4.6 × 10^6^ TUs) (D). (C) Receptor staining of transwell cryosections (7 μm) of S-ALI cultures using lectins SNA, MAI, and MAII is shown; nuclei are stained blue; representative images are shown; scale bars represent 100 μm. (D) Transduced S-ALI cultures were imaged for EGFP fluorescence 4 days after dosing. Representative stitched center-of-transwell images are shown; scale bars represent 500 μm. EGFP fluorescence from transduced S-ALI cultures was also quantified; each data point represents a biological replicate (n = 2); bar represents the median.

To assess the relative affinity of the F/HN pseudotype for human α2,3 and α2,6 glycans, we exploited the hemagglutination property of the HN glycoprotein. The ability to agglutinate red blood cells (RBCs) (hemagglutination) is a well-established property of sialic-acid-binding viruses, employed to identify inhibitors of viral hemagglutination.^30^^,^^31^ As a comparator in these experiments, we also included the LV pseudotype HA Rostock (Figure 6A) derived from influenza A virus, which also uses sialic acid as a receptor for infection of the lung.^13^^,^^14^ We first compared LV.F/HN and LV.HA Rostock for transduction of B-ALI (7.5 × 10^6^ TUs) and SmallAir cultures (4.7 × 10^6^ TUs) and imaged 14 days later. Quantification of % EGFP fluorescence showed that the F/HN pseudotype was more efficient (>300-fold) at transducing B-ALI cultures (4.24% versus 0.03%; p < 0.0001) and also more efficient at transducing SmallAir cultures (0.15% versus 0%; p = 0.0245; Figure 6B). In addition, we transduced human surfactant-ALI (S-ALI) cultures, which model aspects of human lung parenchyma.^32^^,^^33^ Lectin staining of these S-ALI cultures was only positive for MAI lectin, indicating the exclusive presence of the human α2,3 subtype of sialylated glycan (Figure 6C). The LV.F/HN transduced the S-ALI cultures with ∼30-fold greater efficiency than LV.HA Rostock, indicating an increased efficiency for LV.F/HN to use the human α2,3 subtype as a receptor for transduction (Figure 6D).

We next compared the ability of human α2,3 subtype and α2,6 sialylated glycans to inhibit hemagglutination mediated by the F/HN and HA Rostock pseudotypes. In two independent experiments, using different batches of RBCs, vectors were first incubated with serial dilutions of sialylated (or non-sialylated; asialo) glycans to allow binding, prior to mixing with RBCs. Hemagglutination was determined by visual inspection (Table 1). As expected, there was no inhibition of hemagglutination with the asialo glycan, consistent with the observation that both the F/HN and HA Rostock pseudotypes depend on sialic acid for transduction. Hemagglutination by both F/HN and HA Rostock pseudotypes was inhibited, however, following incubation with the (human subtype of) α2,3 sialylated glycans (LSTd and 3ʹ-Sialyl-LacNAc), but not by equivalent α2,6 sialylated glycans (LSTc and 6ʹ-Sialyl-LacNAc). These data indicate that both F/HN and HA Rostock pseudotypes bind the α2,3 glycans with a greater affinity than α2,6. Furthermore, compared with HA Rostock, a much lower concentration of LSTd (16-fold less) was required to inhibit hemagglutination by F/HN. Altogether, this suggests the F/HN pseudotype has a greater affinity for binding glycans of the human α2,3 subtype.

**Table 1:** The relative affinity of F/HN and HA Rostock pseudotyped LV for binding of sialylated glycans, using inhibition of hemagglutination

Glycan				Inhibition of hemagglutination (pMol per reaction^a^)	
				Lentiviral pseudotype	
Name	Structure	Lectin	Linkage	F/HN	HA Rostock
6ʹ-Sialyl-LacNAc		SNA	α2,6	none detected	none detected
LSTc				none detected	none detected
3ʹ-Sialyl-LacNAc		MAI	α2,3	4.5 ± 2.1 × 10^4^	4.5 ± 2.1 × 10^4^
LSTd				2.8 ± 1.3 × 10^3^	4.5 ± 2.1 × 10^4^
Asialo		–	none	none detected	none detected

Hemagglutination of red blood cells (RBCs) by pseudotyped LV was determined in the presence of various glycans. The structure of each glycan is shown following the SNFG system: sialic acid (purple diamond), galactose (yellow circle), N-acetylglucosamine (blue square), and N-acetylgalactosamine (yellow square). The glycosidic linkage, and expected lectin binding (Sambucus nigra agglutinin [SNA] or Maackia amurensis [MAI or MAII]), are also shown for each glycan. A range of concentrations was used to determine the relative potency of each glycan to compete with RBC for LV binding. The minimum pMol glycan per reaction to inhibit hemagglutination is indicated. Values indicate mean pMol required to inhibit hemagglutination, calculated from two independent experiments, with different batches of RBCs (Table S2). For each experiment, both pseudotypes were assayed in parallel following HA titration with each RBC batch. Inhibition was determined by observation of RBC pellets matching control reactions in the absence of vector. The maximum pMol of each glycan investigated was dependent on availability and solubility. LSTc and Asialo glycans were investigated to a maximum of 2.5 × 104 pMol. 6ʹ-Sialyl-LacNAc, 3ʹ-Sialyl-LacNAc, and LSTd glycans were investigated to a maximum of 1.6 × 105 pMol.

a Mean ± standard deviation.

## Discussion

The development of pseudotypes for recombinant LVs is an exciting strategy to tackle the challenges of gene delivery to the lungs, especially the need to efficiently target specific lung cell types. In preparation for translation to the clinic, and to help predict results in the human lung, a variety of models have been used, including *in vitro* cell culture and *in vivo* animal delivery. To have the best chance of predicting success in humans, interpretation of both positive and negative results should be assessed in conjunction with an understanding of the limitations of each model. Given the success of the F/HN pseudotype in several lung and airway models, we sought to investigate the use of sialylated glycans as receptors for F/HN transduction of human airway cells of ALI cultures and considered the implications for translation of this pseudotype to the human lung.

We compared three types of ALI culture and showed that each contained basal, ciliated, goblet, and club cells that varied in abundance. Strikingly, whereas MucilAir and SmallAir cultures exhibited a layer of basal cells that was one to two cells in thickness, B-ALI cultures contained basal cell layers of approximately three cells thickness (Figure 2B), an observation also reported by others when using similar cell-seeding densities and protocols for cell expansion and differentiation.^34^ SmallAir cultures contained relatively few goblet cells compared with the other ALI culture types, in line with observations in more distal (versus proximal) human airway epithelia.^35^ We saw some significant differences in TEER between ALI culture types (Figure 2A), but such TEER variation between cultures generated from bronchial primary cells is well known.^36^^,^^37^ Importantly, the presence of a polarized epithelium for each ALI culture type was confirmed by a lack of transduction following apical administration of LV.VSV-G, a characteristic in line with intact, polarized airway cell cultures.^26^ In contrast, LV.F/HN was efficient at transducing the ALI cultures, particularly the B-ALI culture type (Figures 3A and 3B).

Following the hypothesis that F/HN-mediated transduction is dependent on the availability of sialylated glycans, and to predict performance in human lung, we characterized the subtypes available in human ALI cultures in parallel with human lung tissue. Lectins are widely used to detect sialylated glycans on tissues by specifically binding α2,3 and α2,6 glycosidic linkages. Importantly, we chose to use both MAI and MAII lectins from *Maackia amurensis* to determine the specific subtypes of α2,3 glycans present, since published reports of lectin staining using only one lectin, or a mixture of both, are difficult to interpret. We found negligible MAII staining in all ALI cultures and in human lung, indicating an absence of this subtype of α2,3 glycan. However, the abundance and distribution of MAI and SNA staining (of human α2,3 and α2,6 sialylated glycans, respectively) differed between ALI culture types (Figure 4A). Using co-localization of lectin staining and IHC, there was a robust association of α2,3 with ciliated cells and α2,6 sialylation with non-ciliated cells (Figures 4B and 4C), similar to published reports.^22^ A separate published study, however, showed SNA (α2,6) staining on both ciliated and non-ciliated (goblet) cells.^11^ Although we observed SNA staining specific to non-ciliated cells in B-ALI cultures, ciliated cells were also stained in MucilAir cultures (Figure 4C). Similarly, although MAI staining appeared most concentrated on ciliated cells, non-ciliated cells of B-ALI and MucilAir cultures were also found to express this human α2,3 subtype. Overall, we suggest that, although some published reports may be difficult to interpret, the distribution of subtypes may not be specific to a certain cell type.

Previously, transgene expression following LV.F/HN transduction of human MucilAir cultures was reported,^24^^,^^25^ but the transduced cell types were not identified. Here, we observed a trend for LV.F/HN to target ciliated cells in B-ALI cultures (Figures 3C and 3D), although non-ciliated (basal, goblet, and club) cells were also transduced. While the potential to transduce this range of specialized respiratory epithelium cell types might be expected, given similar observations following *in vivo* delivery to the mouse model,^4^ further investigation into the mechanism underpinning LV transduction of basal cells is required. One theory is that a lack of tight junctions associated with goblet cells could be responsible for increasing access,^38^ although this would not explain why these cultures remain resistant to transduction by LV.VSV-G. In line with the proposed use of α2,3 sialylated glycans as F/HN receptors, LV.F/HN transduction was greatest in B-ALI cultures, which showed an abundant and widespread distribution of human α2,3 glycans (Figure 4A); these cultures were also the most similar to human airways stained in parallel. Despite the diffuse MAI staining pattern observed, transduction of only up to ∼50% of cells (Figure 3B) reminds us that not all MAI-stained glycans appear to facilitate transduction. However, studies on the effects of sialidase cleavage, and competition for binding specific glycans, showed that F/HN transduction was mediated primarily via the human α2,3 subtype. In particular, transduction of B-ALI cultures was significantly reduced (2-fold) after treatment with Sialidase S that preferentially cleaves α2,3 linkages (Figure 5C). The observed residual levels of transduction could be attributed to incomplete removal of α2,3 sialylation by Sialidase S or the partial recovery of α2,3 sialylation during the 4-h incubation with the vector. Alternatively, the residual transduction could be mediated by remaining α2,6 sialylated glycans; this is an interesting possibility, since binding of α2,6 glycans by Sendai virus has also been identified.^18^ Overall, however, there was little evidence for use of α2,6 sialylated glycans as receptors for F/HN transduction after pre-incubation of ALI cultures with MAI lectin and only minimal impact of blocking these receptors with SNA (Figures 5D and 5E). Altogether, this suggests that the F/HN pseudotype uses the MAI bound human subtype of α2,3 sialylated glycan most efficiently, and where α2,6 can be used, they are not preferred.

Lentiviral transduction mediated via the F/HN pseudotype was significantly more efficient than the HA Rostock pseudotype, despite the latter being predicted to use α2,3 sialylated glycans as receptors. In previous studies, both the F/HN^4^ and HA Rostock^12^^,^^13^ pseudotypes transduced the murine lung efficiently, but here, we show that only F/HN transduces human ALI cultures (Figure 6B). This could be due to differences in the ability of these pseudotypes to use the human α2,3 subtype as a receptor. This hypothesis is strongly supported by the greater efficiency of F/HN transduction in S-ALI cultures that exclusively display the human α2,3 subtype (Figure 6D) and also in hemagglutination experiments, where F/HN has a greater (16-fold) affinity for the LSTd glycan of this subtype (Table 1). Interestingly, FPV H7N1, from which the HA Rostock pseudotype is derived, binds α2,3 sialylated glycans of the MAII subtype (GD1a; see Figure S3) with greater affinity (∼7-fold) than Sialyl-3-paragloboside (MAI subtype),^14^ although the relative use of these glycans as receptors for transduction is unknown. Indeed, a potential criticism of this experiment to compare pseudotypes is the overall inefficiency of HA Rostock for cell transduction. However, when we compared transduction efficiencies in a range of cell lines, the difference in efficiency appeared restricted to cells which lacked the MAII subtype of α2,3 (Figures S4 and S5). This suggests that HA Rostock depends on the MAII α2,3 subtype, a finding in line with the FPV affinity. In contrast, the affinity of Sendai virus for Sialyl-3-paragloboside (MAI subtype) is ∼10-fold greater than for GD1a,^18^ suggesting preferential use of the MAI stained human α2,3 subtype as receptors over the MAII subtype. This would, however, need to be investigated in a model abundant in both α2,3 subtypes, such as mouse.

The majority of *in vivo* animal experiments to assess the efficacy and safety of gene transfer, such as in toxicology studies, are performed in rodents (particularly mice); and the translatability of such studies to the human situation is therefore an important consideration. Two previous studies probing the distribution of α2,3 sialylated glycans in the murine lung were only partly successful, since they used either an undefined mixture of MAI and MAII lectins^11^ or only MAII.^19^ Here, we used both MAI and MAII individually to demonstrate an abundance of both α2,3 subtypes in the murine airway and lung (Figure S2), but this is in contrast with the human airway (Figure 4A). Furthermore, our findings indicate differences in the ability of pseudotypes to target the different α2,3 subtypes. Altogether, this highlights some significant problems in extrapolating positive results in murine lung to predict results in humans. In order to evaluate vectors targeting sialylated glycans *in vivo*, models such as ferret or pig, regularly used to inform the infectivity and transmission potential of influenza viruses in humans,^39^^,^^40^ could be more useful.

In conclusion, this study demonstrates the importance of selecting an appropriate model in which to assess gene transfer efficiency. Careful assessment of the distribution of different subtypes of sialylated glycans, and the transduced cell types, has confirmed the utility of human ALI models to predict gene transfer success in the human lung. The F/HN pseudotype has previously been shown to efficiently transduce the murine lung, but the receptors used were unclear. Given that we have identified a preference for F/HN to bind the human a2,3 subtype, we suggest that this could also be a subtype used by F/HN for efficient transduction in the murine lung. Importantly, the ability to efficiently use the human α2,3 subtype, which is abundant throughout the human lung, predicts that the F/HN pseudotype could facilitate efficient gene delivery to the human lung for treatment of human lung diseases.

## Materials and methods

### Lentiviral vectors

Recombinant replication-defective, self-inactivating HIV-based lentiviral vectors were produced using plasmids obtained from Oxford Biomedica (UK): Rev (pOXB-REV) and Gag-pol (pOXB-HSGP) packaging plasmids in combination with a vector genome plasmid encoding EGFP under the transcriptional control of either a cytomegalovirus (CMV) (pOXB-HVCG) or hCEF (pOXB-hCEF-EGFP) promoter.^41^ Two plasmids were used for each pseudotype: for F/HN, pOXB-CMV-coFct4 and pOXB-CMV-SIV-coHN (sequences derived from Kobayashi et al.^42^) and, for HA Rostock, pOXB-Vitro2-H7rN1pr and pOXB-RKH-M2 (Patel et al.^12^). Vectors were produced at the 500-mL scale using the LV-MAX Lentiviral Production System (Gibco) according to manufacturer’s instruction. Vector production was initiated via transfection of a total of 1,250 μg plasmid at the following mass ratios (Rev:Gag-pol:vector genome:pseudotype1:pseudotype2): LV.F/HN (11:14:45:9:9) and LV.HA Rostock (20:83:83:50:8). Viral supernatant was harvested 48 h post-transfection, purified using anion exchange chromatography (1.72 mL membrane volume; Mustang QXT Anion Exchange membranes from Pall, Life Sciences, Portsmouth, UK) and concentrated by tangential flow filtration (115 cm^2^ modified polyethersulfone 500-kDa hollow fiber membrane from Repligen, CA). Vectors were formulated in TSSM (20 mM Tromethamine, 100 mM NaCl, 10 mg/mL sucrose, and 10 mg/mL mannitol)^43^ and stored in single-use aliquots at −80°C prior to use. LV.VSV-G, which served as a negative control, was produced using the same Gag-Pol, Rev, and vector genome plasmids and an alternate VSV-G pseudotyping plasmid and was a gift of Oxford Biomedica (UK).

Functional titers (TUs/mL) were determined using a qPCR assay to detect integrated lentiviral genomes 72 h after transduction of the HEK293-derived LV-MAX cells. Primers targeting a region of the integrated vector genome sequence—posttranscriptional regulatory element of woodchuck hepatitis virus (WPRE) sequence (forward: 5ʹ-TGGCGTGGTGTGCACTGT-3ʹ; reverse: 5ʹ-CCCGGAAAGGAGCTGACA-3ʹ; probe: 5ʹ-FAM-TTGCTGACGCAACCCCCACTGG-TAMRA-3ʹ) and an endogenous control (hCFTR [human Cystic Fibrosis Transmebrane conductance Regulator], forward: 5ʹ-CTTCCCCCATCTTGGTTGTTC-3ʹ; reverse: 5ʹ-TGACAGTTGACAATGAAGATAAAGATGA-3ʹ; probe: 5ʹ-VIC-TGTCCCCATTCCAGCCATTTGTATCCT-TAMRA-3ʹ)—were used to quantify copies against a DNA standard curve created by diluting known copies of a plasmid DNA molecule containing the WPRE and hCFTR PCR amplicons.

### Air-liquid interface cultures

The B-ALI cultures were generated using HBEC obtained from Lonza (Basel, Switzerland CC-2540S), following expansion and differentiation using the PneumaCult culture system (STEMCELL Technologies, Vancouver, Canada) on 6.5-mm-diameter (0.33 cm^2^), 0.4-μm-pore transwells (Corning). Epithelix cultures (SmallAir and MucilAir) were obtained fully differentiated (>45 days post-airlift) from Epithelix Sarl (Geneva, Switzerland). ALI cultures were maintained using the recommended media, which was exchanged in the basal chamber every 48–72 h and subjected to PBS washing (20 min) once per week to remove excess mucus. All differentiated ALI cultures (8 weeks post-B-ALI airlift) were treated or transduced apically, 24 h following washing, with the appropriate dose of vector in 100-μL TSSM buffer for 3.5 h at 37°C. After 14 days, treated cultures were imaged to detect EGFP under identical settings using the EVOS FL Auto 2 Imaging System (Invitrogen) and analyzed to determine percentage area EGFP above fixed background threshold performed using ImageJ (NIH).

S-ALI cultures were generated from H441 cells as described by Munis et al.^33^ We seeded 2.5 × 10^4^ cells on 6.5-mm-diameter (0.33 cm^2^), 0.4-μm-pore transwells (Corning) and cultured submerged in RPMI 1640 (A1049101) supplemented with 10% fetal calf serum (FCS) (Sigma) and 1% PenStrep (Gibco). At 72 h, the cultures were air lifted and media exchanged for S-ALI polarization medium (RPMI 1640 supplemented with 4% FCS, 1% insulin-transferrin-selenium [ITS] [Gibco], 1% PenStrep, and 1 μM dexamethasone [Sigma]). Cultures were transduced at 14 days post-airlift and imaged as above 4 days after vector delivery.

### TEER

Measurements of TEER were performed using Millicell 392 ERS-2 Voltohmmeter (Milipore) according to manufacturer’s instructions. Briefly, cultures were submerged and equilibrated in 200 μL apical and 500 μL basolateral Dulbecco’s phosphate-buffered saline (DPBS) for 15 min at room temperature prior to measurement with the “chopstick” electrodes. Resistance values were normalized by subtracting the TEER observed from transwells wetted with the appropriate culture media but without seeded cells and adjusted to the culture area of the transwells for presentation as Ω.cm^2^.

### Sialidase and lectin treatments

The ALI cultures were apically treated with 25 mU Sialidase A (*Arthrobacter ureafaciens*, AdvanceBio GK80040, Agilent) or Sialidase S (AdvanceBio GK80021, Agilent) diluted in reaction buffer B provided (5×, 250 mM sodium phosphate [pH 6]) to 50 μL for 1 h at 37°C prior to fixation (lectin staining) or transduction. Alternatively unconjugated lectins from either SNA or MAI or MAII obtained from Vector Laboratories (Burlingame, CA) were diluted to 100 μg/mL in DPBS and 100 μL apically applied to cultures for 2 h at 37°C prior to transduction.

### Cryosection immunohistochemistry and lectin staining

Fixed cryosections of human lung were a kind gift from Dr. Gerry McLachlan (Roslin Institute Edinburgh, UK), obtained following approval by Oxford Central University Research Ethics Committee (CUREC) (R59182) and stored under the University of Oxford HTA license 12,217. Approximately 7-μm cryosections of ALI cultures on transwells were generated (CryoStar NX50 Thermo Fisher Scientific) after fixation and dehydration of cells (20 min 4% paraformaldehyde in PBS [pH 7]; >1 h 30% sucrose) and embedding of transwells in optimal cutting temperature (OCT) compound. For immunohistochemistry, cryosections were permeabilized and blocked for 1 h using 1% BSA and 5% normal goat serum (NGS) in PBST (PBS with 0.1% Triton X-100) and sections incubated with the relevant primary antibodies diluted in blocking buffer at 4°C overnight. Primary antibody binding was detected using goat secondary antibody raised against respective primary species immunoglobulin G (IgG) conjugated to Alexa Fluor 594 (rabbit A-11012 and mouse A-11005, Invitrogen, Thermo Fisher Scientific) diluted in blocking buffer and applied for 1 h at room temperature. The following primary antibodies were used to stain for cell types in ALI cultures: anti-cytokeratin 5 for basal cells (Ab52635 Abcam, Cambridge, UK), anti-β-tubulin for ciliated cells (MAB3408 Chemicon International, Temecula, CA), anti-mucin 5Ac for goblet cells (Ab212636 Abcam, Cambridge, UK), and anti-CC10 for club cells (sc365992 Santa Cruz Biotechnology, Dallas, Texas). To control for non-specific sialylated receptor binding by lectins, untreated cryosections were incubated with sialidase A (0.5 U/mL; 37°C overnight).

Staining using fluorescein isothiocyanate (FITC) conjugated or biotinylated lectins was performed on cryosections, untreated or treated with sialidase A (in culture or untreated control cryosections), following antigen retrieval in freshly prepared citrate buffer (pH 6) (15 min at 98°C). Sections were then blocked in Tris-buffered saline (TBS)/1% BSA for 45 min and the Streptavidin/Biotin Blocking kit (Vector Laboratories) according to manufacturer’s instructions. FITC-conjugated or biotinylated lectin from SNA or biotinylated MAI and MAII from Vector Laboratories were used to detect sialylated receptors, at 10 μg/mL in TBS/1% BSA applied to sections overnight at 4°C. Biotinylated lectin binding was detected using streptavidin conjugated to Alexa Fluor 594 (Invitrogen, Thermo Fisher Scientific) diluted in TBS/1% BSA, applied for 1 h at room temperature. Sections were mounted using ProLong Gold Antifade Mountant with DAPI (P36935, Invitrogen, Thermo Fisher Scientific) and imaged the following day using EVOS Auto 2 FL scanning microscope.

### Hemagglutination inhibition

Adult chicken red blood cells (RBCs), preserved in Alsever’s solution (FB010AP, TCS Biosciences Buckingham, UK), were washed with PBS immediately prior to use. Prior to each inhibition assay, the minimum concentration of each LV required to hemagglutinate the RBC used, also known as the HA unit,^30^ was determined by HA titration (Table S1). Briefly, serial dilutions of each LV preparation in 50 μL TSSM were made prior to addition of 50 μL of 1% RBC (final volume 0.5%); the last well in which complete hemagglutination is observed contains one HA unit. Hemagglutination was determined by visual inspection for absence of RBC pellet in 96-well U-bottom plates after 45 min incubation of RBC and LV mixtures at room temperature, example shown (Figure S6). All glycans were obtained from either Dextra (Reading, UK)—3ʹ-Sialyl-LacNAc (SLN302), 6ʹ-Sialyl-LacNAc (SLN306), and LSTc (SLN506)—or from Elicityl (Crolles, France), LSTd (GLY083), and Galacto-N-Neopentaose (GLY024). For inhibition assays, 2-fold serial dilutions of each glycan were prepared in DPBS and incubated at 37°C for 1 h with one HA unit of LV in a fixed volume of TSSM per assay, prior to RBC addition to determine hemagglutination as before.

### Statistics

All statistical tests were carried out using GraphPad Prism 7 software. Tests performed and respective p values are detailed in figure legends.

## Data availability

The authors confirm that the data supporting the findings of this study are available within the article and its supplemental materials.

## Supplemental information

Document S1. Figures S1–S6 and Tables S1 and S2

Document S2. Article plus supplemental information
