# Supplementary material for: Sendai F/HN pseudotyped lentiviral vector transduces human ciliated and non-ciliated airway cells using α 2,3 sialylated receptors
Source: Mol Ther Methods Clin Dev. 2022 Jul 6;26:239–52. doi: 10.1016/j.omtm.2022.07.002 (PMC9304433; doi:10.1016/j.omtm.2022.07.002)
Supplement: Document S1. Figures S1–S6 and Tables S1 and S2 [file mmc1.pdf]

OMTM, Volume *26*

Supplemental information

Sendai F/HN pseudotyped lentiviral vector transduces human ciliated and non-ciliated airway cells using α 2,3 sialylated receptors

Rosie J. Munday, Tiziana Coradin, Rachael Nimmo, Yatish Lad, Stephen C. Hyde, Kyriacos Mitrophanos, and Deborah R. Gill

Supplemental Information

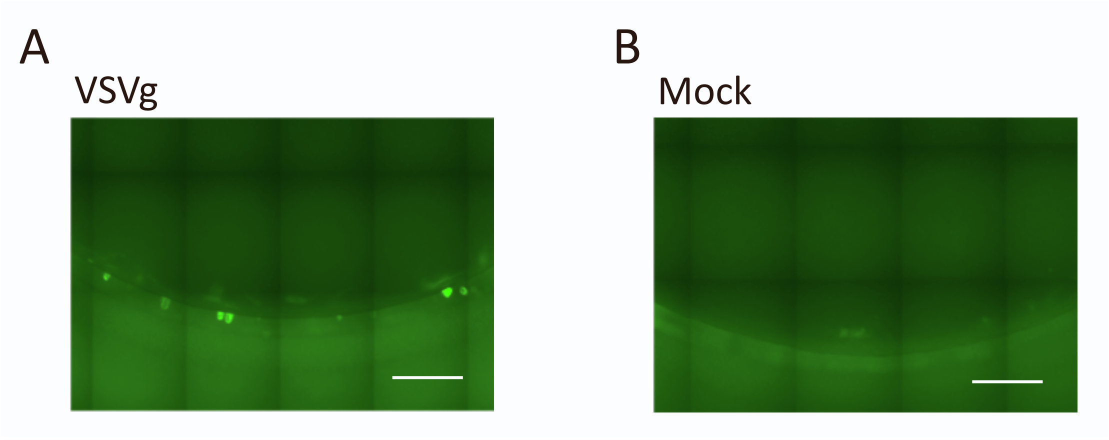

Supplementary Figure S1: EGFP expression following apical treatment with LV.VSV-G is restricted to the edge of ALI culture

Stitched whole-transwell images of ALI cultures captured using the EVOS FL Auto 2 Imaging System (Invitrogen) were analysed to determine the percentage area of EGFP above a fixed background threshold using Image J (NIH). The region of interest was set to exclude the edge of ALI culture transwells where disruption may allow access to basolateral receptors and could explain low level transduction by LV.VSV-G

A) Representative image of native EGFP expression detected at the edge of ALI culture transwells 14 days after apical delivery of $7.5x10^7$ TU of LV.VSV-G expressing EGFP (n=2 biological replicates; B-ALI n=4 donors; MucilAir and SmallAir n=2 donors; see **Figure 3A** for centre-of-transwell images).

B) Image representative of mock-treated ALI cultures also shown for comparison; scale bar represents 500μM.

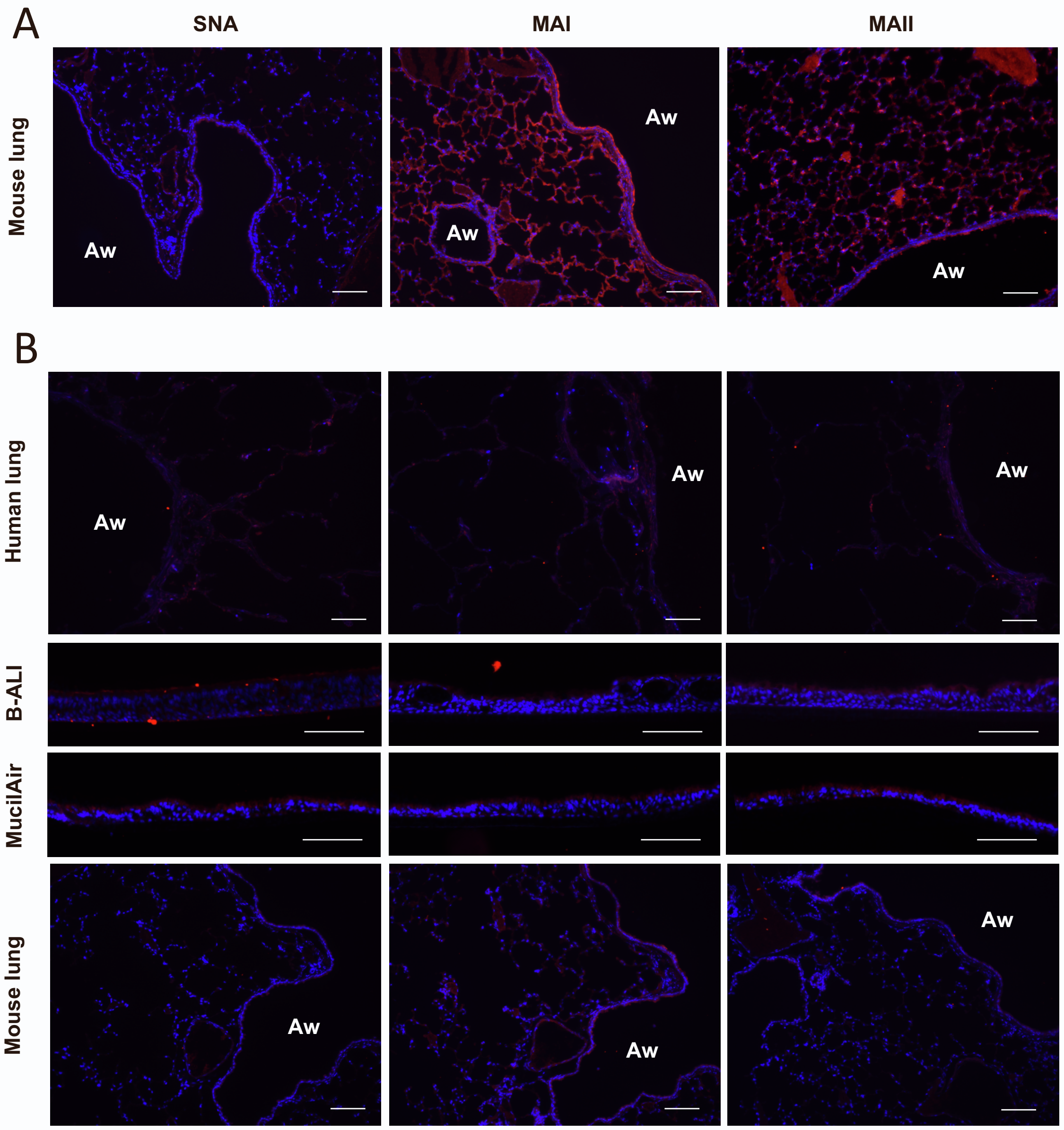

Supplementary Figure S2: Lectin staining of mouse lung cryosections and Sialidase A treated negative control human lung, ALI and mouse lung cryosections.

Cryosections of murine lung tissue were stained with lectins and imaged in parallel with the human lung and ALI cultures shown in Figure 4A. Staining using FITC-conjugated or biotinylated lectins was performed on cryosections of mouse lung in parallel with human lung and ALI culture transwells. Prior to antigen retrieval additional control sections were pre-treated with Sialidase A (0.5U/ml 37°C) to cleave all sialic acid prior to staining. Minimal levels

of fluorescence were detected from all pre-treated tissue and transwell sections. In contrast, ubiquitous staining of the murine airway and lung with MAI and MAII lectins was detected from untreated tissue.

A) Lectin staining (SNA, MAI and MAII) of cryosections generated from murine lung (BALB/C mice).

B) Cryosections from human lung, ALI cultures (B-ALI and MucilAir) and murine lung, that were all pre-treated with Sialidase from *Arthrobacter ureafaciens* (Sialidase A; neuraminidase) to cleave all sialic acid prior to staining, were also imaged in parallel to confirm staining is specific to sialylated glycans. Representative images shown, nuclei are stained blue (DAPI). Aw: airway, scale bar represents 100um.

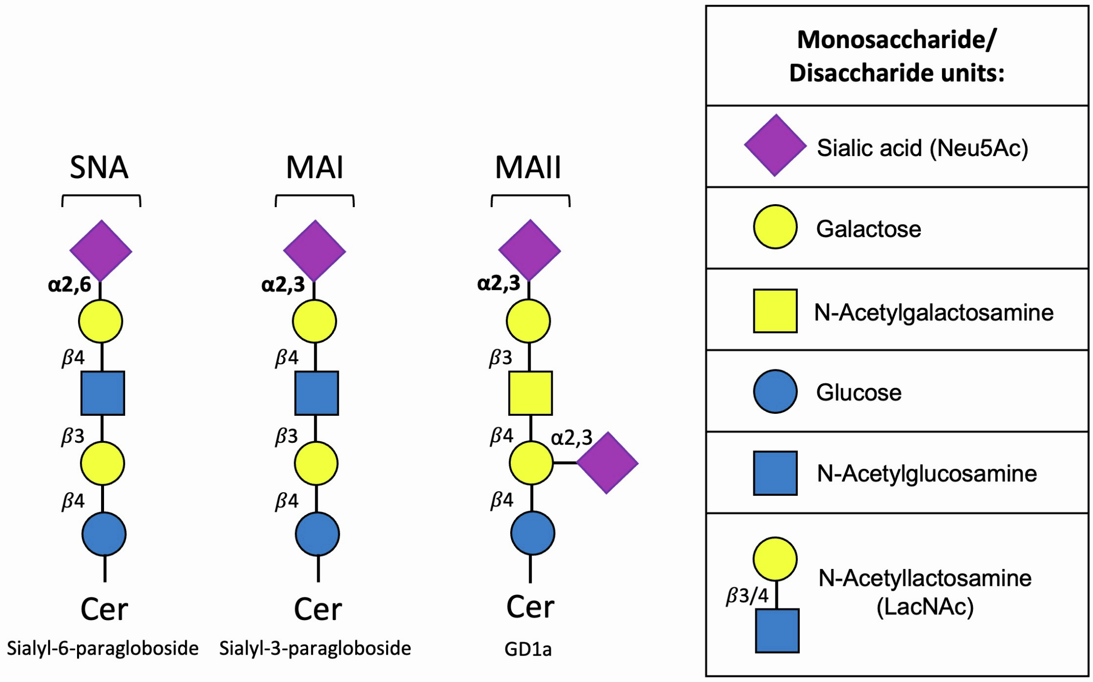

Supplementary Figure S3: The structure of common sialylated glycans found on lipids and used in traditional solid phase binding assays.

Examples of glycolipids employed to investigate virus binding to neolacto-series (Sialyl-6-paragloboside or Sialyl-3-paragloboside) or ganglio-series (GD1a) glycans. Cer; ceramide. Predicted lectin binding also shown. Monosaccharides represented following the Symbol Nomenclature for Glycans system.

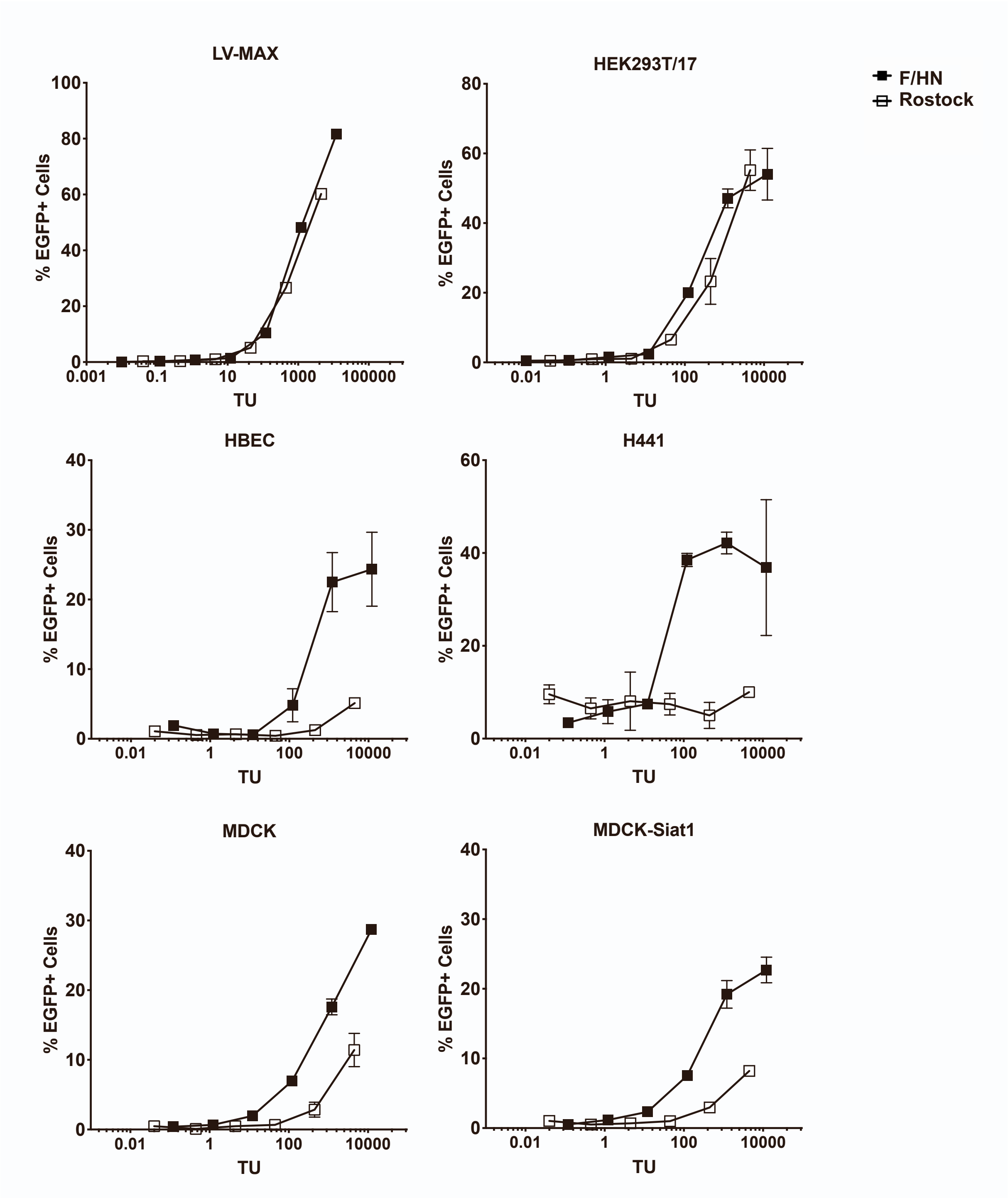

Supplementary Figure S4: Investigating the relative efficiency of F/HN and HA Rostock in different cells.

LV-MAX cells were transduced with dilutions of LV.F/HN and LV.HA Rostock prepared in serum free media in parallel with adherent cultures of Human Embryonic kidney (HEK) 293T cells (HEK293T/17 CRL-11268; American Type Culture Collection (ATCC), HBEC from a

single donor (1; during expansion), H441 cells (HTB-174Manassas, USA); Madin-Darby Canine Kidney (MDCK (NBL-2) CCL-34; ATCC); Madin-Darby Canine Kidney cells stably transfected with human α2,6-sialytransferase 1 (SIAT1) cDNA (MDCK-SIAT1; ECACC 05071502). Approximately 6hrs prior to transduction, 16000 cells were seeded per 96-well in 50ul serum free media. Two hours after the addition of LV (50ul prepared in TSSM), 200ul media was added and EGFP expression determined by fluorescence-activated cell sorting (FACS) after 40hours. These adherent cells were maintained in either Dulbecco's Modified Eagle's Medium (DMEM; Life Technologies) (HEK293T & MDCK-SIAT1), Eagle's Minimum Essential Medium (EMEM; ATCC) (MDCK), or RPMI1640 (H441), supplemented with 100 U/mL penicillin plus 100 mg/mL streptomycin (Sigma) and 2mM L-Glutamine (Life Technologies), as well as 10% Foetal Bovine Serum for culture prior to transduction (FBS; Sigma or ATCC).

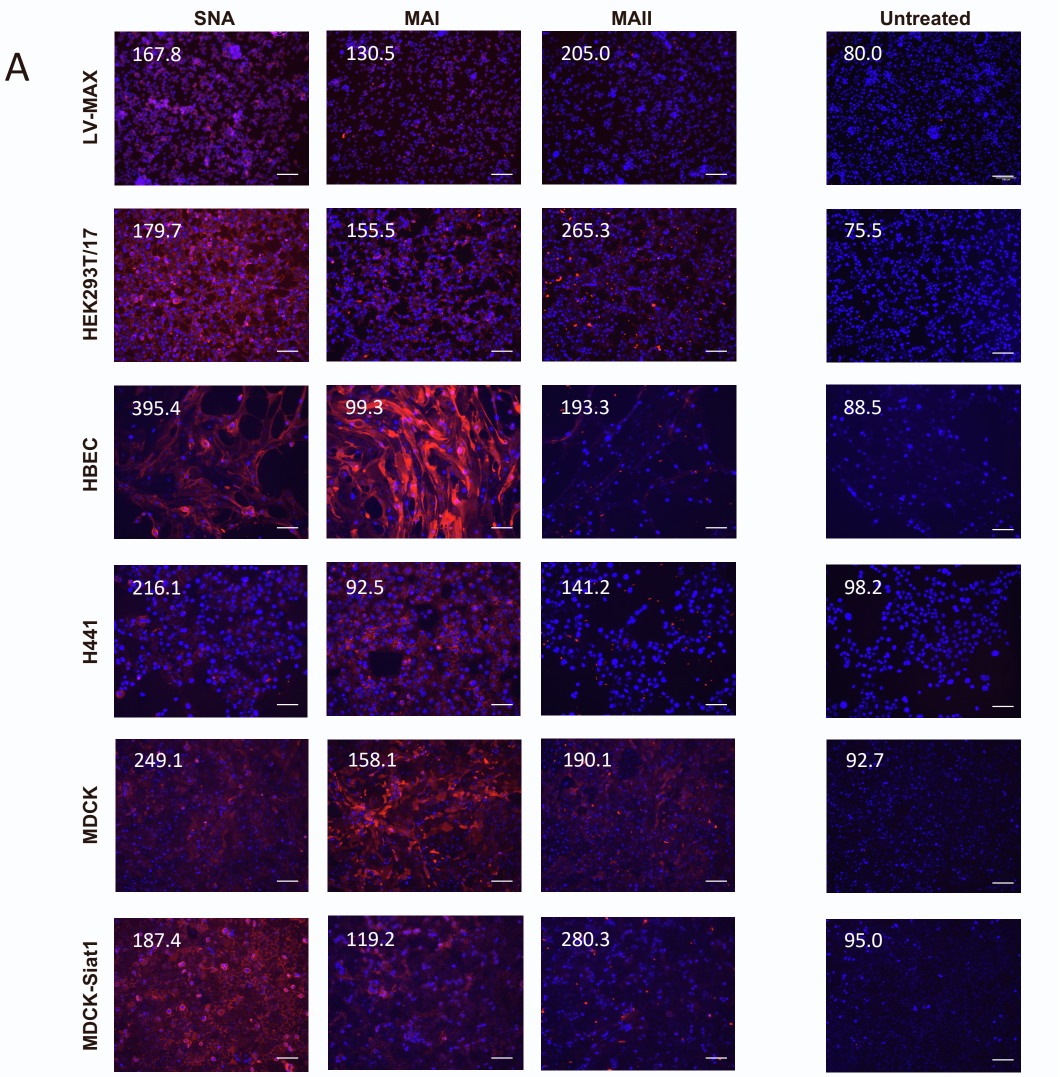
A
SNA
MAI
MAII
Untreated
LV-MAX
HEK293T/17
HBEC
H441
MDCK
MDCK-Siat1
167.8
130.5
205.0
80.0
179.7
155.5
265.3
75.5
395.4
99.3
193.3
88.5
216.1
92.5
141.2
98.2
249.1
158.1
190.1
92.7
187.4
119.2
280.3
95.0

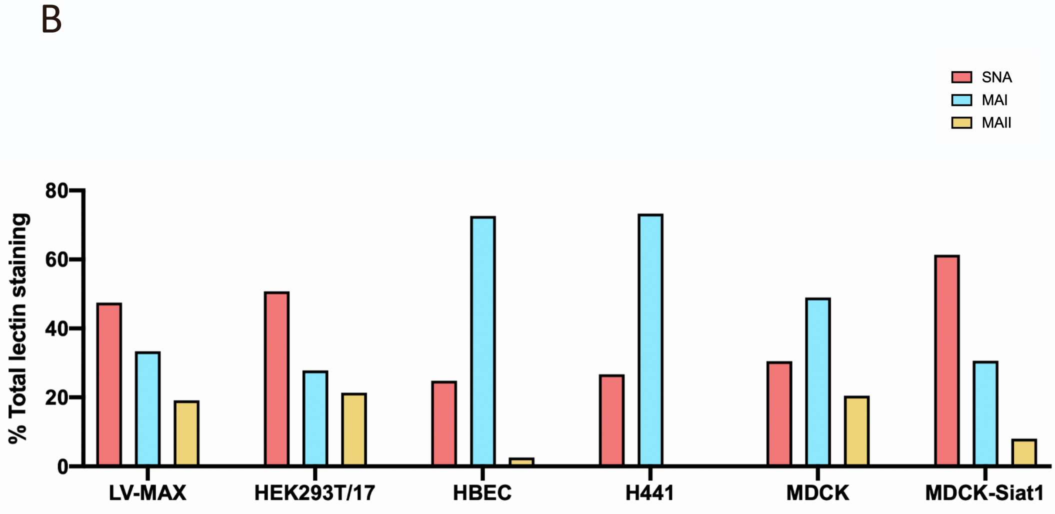
B
SNA
MAI
MAII
% Total lectin staining
0
20
40
60
80
LV-MAX
HEK293T/17
HBEC
H441
MDCK
MDCK-Siat1

Supplementary Figure S5: Use of lectins to investigate the relative availability of sialylated glycan subtypes between different cells.

In parallel with transductions (**Supplementary Figure S4)**, cells seeded to 24-well plates at an equivalent density (96000/well) were stained using lectins *in situ* to determine the relative availability of sialylated glycans. Cells were fixed (4% PFA 20 min), blocked using TBS/1% BSA for 45min, and a Streptavidin/Biotin Blocking kit (Vector Laboratories) according to the manufacturer's instructions. Biotinylated lectin from *Sambucus nigra* agglutinin (SNA) or biotinylated *Maackia amurensis* lectins I and II (MAI and MAII) from Vector Laboratories were then used to detect sialylated receptors, at 10μg/mL in TBS/1% BSA applied to cells overnight at 4°C. Lectin binding was detected using Streptavidin conjugated to Alexa Fluor 594 (Invitrogen, Thermo Fisher Scientific) diluted in TBS/1% BSA, applied for 1hr at room temperature. Sections were counter stained using DAPI prior to imaging in 100ul TBS (EVOS Auto 2 FL scanning microscope). The fluorescence intensity from the RFP channel was determined using mean grey value quantification in image J and images captured with the same magnification and settings. To determine the relative availability of receptors for each cell type, values calculated for each lectin were subtracted from the levels of autofluorescence detected from untreated cells (**Supplementary Figure S5A).** These values were then represented as a % of total lectin staining for each cell type **(Supplementary Figure S5B).**

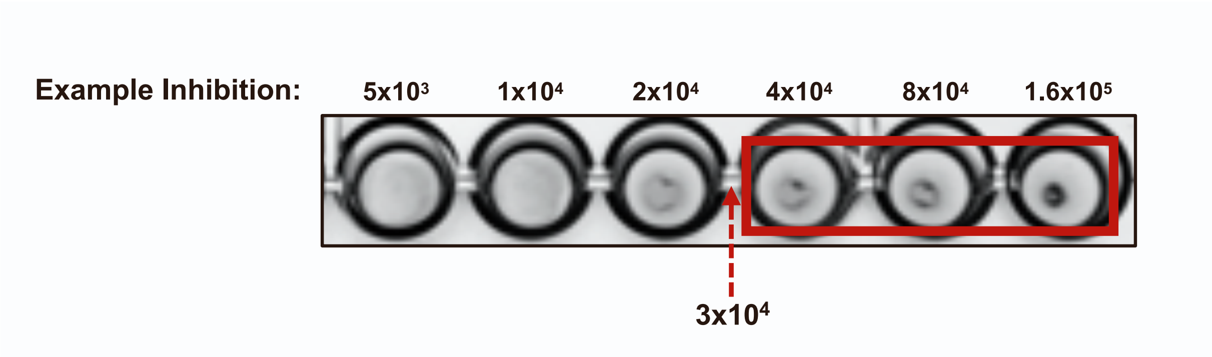

Supplementary Figure S6: An example of hemagglutination inhibition

Inhibition of hemagglutination (red box) is shown, from which the lowest pMol glycan required to hemagglutinate (RBC batch B, 3'-Sialyl-LacNAc, F/HN) is shown.

Supplementary Table S1: **Dilution of pseudotyped LV required to agglutinate a 100ul reaction of 0.5% volume RBC**

	HA unit (dilution factor)	
**RBC batch**	**A**	**B**
**LV.F/HN**	2560	5120
**LV.HA Rostock**	2560	5120

Supplementary Table S2: Concentration of glycan required to inhibit the hemagglutination of two different batches of RBC

Values used to determine mean pMol for each glycan and pseudotype (Table 1).

	**Inhibition of Hemagglutination (pMol per reaction)**			
	**Lentiviral Pseudotype**			
	**F/HN**		**HA Rostock**	
**RBC Batch**	**A**	**B**	**A**	**B**
6'-Sialyl-LacNAc	None Detected	None Detected	None Detected	None Detected
LSTc	None Detected	None Detected	None Detected	None Detected
3'-Sialyl-LacNAc	60000	30000	60000	30000
LSTd	3750	1875	60000	30000
Asialo	None Detected	None Detected	None Detected	None Detected
